# New Trends and Most Promising Therapeutic Strategies for Epilepsy Treatment

**DOI:** 10.3389/fneur.2021.753753

**Published:** 2021-12-07

**Authors:** Antonella Riva, Alice Golda, Ganna Balagura, Elisabetta Amadori, Maria Stella Vari, Gianluca Piccolo, Michele Iacomino, Simona Lattanzi, Vincenzo Salpietro, Carlo Minetti, Pasquale Striano

**Affiliations:** ^1^Pediatric Neurology and Muscular Diseases Unit, IRCCS Istituto Giannina Gaslini, Genoa, Italy; ^2^Department of Neurosciences, Rehabilitation, Ophthalmology, Genetics, Maternal and Child Health, University of Genoa, Genoa, Italy; ^3^Department of Functional Genomics, Center for Neurogenomics and Cognitive Research, Vrije Universiteit, Amsterdam, Netherlands; ^4^Unit of Medical Genetics, IRCCS Istituto Giannina Gaslini, Genoa, Italy; ^5^Department of Experimental and Clinical Medicine, Neurological Clinic, Marche Polytechnic University, Ancona, Italy

**Keywords:** anti-seizure medications, epilepsy, genetics, inflammation, precision medicine

## Abstract

**Background:** Despite the wide availability of novel anti-seizure medications (ASMs), 30% of patients with epilepsy retain persistent seizures with a significant burden in comorbidity and an increased risk of premature death. This review aims to discuss the therapeutic strategies, both pharmacological and non-, which are currently in the pipeline.

**Methods:** PubMed, Scopus, and EMBASE databases were screened for experimental and clinical studies, meta-analysis, and structured reviews published between January 2018 and September 2021. The terms “epilepsy,” “treatment” or “therapy,” and “novel” were used to filter the results.

**Conclusions:** The common feature linking all the novel therapeutic approaches is the spasmodic rush toward precision medicine, aiming at holistically evaluating patients, and treating them accordingly as a whole. Toward this goal, different forms of intervention may be embraced, starting from the choice of the most suitable drug according to the type of epilepsy of an individual or expected adverse effects, to the outstanding field of gene therapy. Moreover, innovative insights come from *in-vitro* and *in-vivo* studies on the role of inflammation and stem cells in the brain. Further studies on both efficacy and safety are needed, with the challenge to mature evidence into reliable assets, ameliorating the symptoms of patients, and answering the challenges of this disease.

## Introduction

Epilepsy is the enduring predisposition of the brain to generate seizures, a condition that carries neurobiological, cognitive, psychological, and social consequences (1). Over 50 million people worldwide are affected by epilepsy and its causes remain partially elusive, leaving physicians, and patients an unclear insight into the etiology of the disease and the best treatment approach (2). Over than 30% of individuals do not respond to common anti-seizure medications (ASMs) and are addressed to as “drug-resistant,” a term which the International League Against Epilepsy (ILAE) applies to those patients who do not respond to the combination of two appropriately chosen and administered ASMs (3, 4). Hence, a great deal of responsibility laid upon the research and development of innovative pharmacological and non-pharmacological approaches given a targeted approach, aiming at improving the symptoms of patients and their quality of life (QoL), together with that of the caregivers.

As several investigations are currently in progress, this review aimed to discuss the novel therapeutic insights, with the hope they may establish as turning points in the treatment of patients in the next few years.

## Methods

A search on PubMed, Scopus, and EMBASE databases using the terms “epilepsy,” “treatment” or “therapy,” and “novel” was conducted. The search covered the period between January 2018 and September 2021. Existing literature was reviewed, including both experimental and clinical studies, meta-analysis, and topic reviews summarizing the most up-to-date researches. Only studies published in English were reviewed.

## Precision Medicine

Precision medicine (PM) is an outstanding approach tended to use the genetics, environment, and lifestyle of individuals to help determine the best “way” to prevent or treat disease (5). It embeds a holistic evaluation, assessing not only the effect of an own condition but also that of treatment (6). Precision medicine is endorsed in epilepsy management for many decades, as in the clinical practice ASMs are selected after a careful and pointful evaluation of seizure types of patients, their epilepsy syndrome, comorbidities, concomitant drugs, and expected vulnerability to specific adverse events (AEs) (7). Discoveries and progress in genetics have provided the strongest basis for PM: as more and more genes are being identified as disease-causing, hope has grown on possible targeted approaches (6). An “ideal” therapy would be able to both relieve symptoms and reverse the functional alterations caused by specific genetic mutations. This firstly implies identifying putative disease-causing genes and, secondly, the specific functional alterations caused by the pathogenic variants. Lastly, it should have been demonstrated that therapeutic intervention may modify the effect caused by the mutation.

The ketogenic diet (KD) used to treat glucose transporter 1 (GLUT1) deficiency syndrome is probably the best example of PM applied to epilepsy. In GLUT1 patients the uptake of glucose into the brain is impaired because of the *SLC2A1* mutation, hence, the KD provides neurons with an alternative source of energy, compensating for the consequences of the metabolic defect (8). Another clear application of a PM-based approach is the avoidance of those drugs which may cause worsening of seizures by exasperating the underlying molecular defect, i.e., sodium channel blockers must be avoided in patients with Dravet syndrome (DS) carrying loss-of-function mutations in the sodium voltage-gated channel alpha subunit 1 (*SCN1A*). Another one is memantine for the treatment of GRIN-related disorders due to gain-of-function mutations in the NMDA receptor (8–11) or quinidine and retigabine for epilepsies caused by potassium channels genes mutations (*KCNT1* and *KCNQ2*) (6, 12). In epileptic encephalopathies (EE), it would be also of interest to investigate the effect of a PM treatment on cognitive function, to that targeting a specific gene mutation and abolishing related epileptic activity may result in improved cognitive functions (10).

Precision medicine may prove complex, as the same mutation may cause quite different clinical phenotypes; moreover, additional genetic variants may contribute to modifying a phenotype. Again, wide-genome variations or even the epigenome may influence the resulting expression of pathogenic variants (5).

Nowadays, evidence indicates PM may be applied to individuals with both rare and common forms of epilepsy, and, consequently, drug development is increasingly being influenced by PM approaches. Although extensive research focuses on genome-guided therapies, important opportunities also derive from immunosuppressive therapies and neuroinflammation-targeting treatments (2, 13). The identification of cellular and molecular biomarkers would possibly allow clinicians to have early prediction markers of a disease and its progression. Additionally, it could lead to the development of unique models to cost-effectively screen treatments and also decrease the costs of clinical trials through better patient selection (14).

## Novel Mechanisms of Anti-Seizure Medications

Many medications are currently under study in clinical practice, ranging from those with a mechanism similar to that of well-known ASMs, like the GABA-A receptor agonists, to those with novel mechanisms such as the stimulation of melatonin receptors. Moreover, some drugs are yet known medications, previously used for other indications; while a large group remains orphan of a well-comprised mechanism of action (6). It is in this perspective, that the wider term ASMs should be addressed, aiming at referring to the large heterogeneity of action mechanisms nowadays available to counteract seizures.

### Cannabidiol

In 2018, the Food and Drug Administration (FDA) approved the first-in-class drug derived from the cannabis plant. Although the precise mechanism by which the cannabidiol (CBD) exerts its anti-seizure effects is still poorly known, it seems not to act through interaction with known cannabinoid receptors (15), but holds an affinity for multiple targets, resulting in the reduction of neuronal excitability which is relevant for the pathophysiology of the disease (16, 17).

Cannabidiol is approved for the treatment of seizures in children with DS or Lennox-Gastaut syndrome (LGS) aged 2 years or older, based on three pivotal phases 3 trials (12). In 2019 CBD gained approval in Europe in conjunction with clobazam (CLB), based on clinical trial data showing that the combination of both CBD and CLB resulted in greater efficacy outcomes (16).

The first clinical trial (17) included 120 patients with DS aged between 2 and 18 years. The median frequency of convulsive seizures decreased from 12.4 to 5.9 per month, as compared with a decrease from 14.9 to 14.1 per month with the placebo. Furthermore, 43% of patients in the active arm and 27% in the placebo group showed at least a 50% reduction in the convulsive-seizure frequency. Overall, 62% of patients under CBD did gain at least one category at the seven-category Caregiver Global Impression of Change scale, as compared to 34% in the placebo group. Five percent of patients under CBD became seizure-free, while none in the placebo group did.

Another randomized, double-blind, placebo-controlled, trial (18) included 171 LGS patients aged between 2 and 55 years and measured the reduction in drop-seizures. The median percentage reduction was 43.9% in the CBD group and 21.8% in the placebo group. In 2018, Devinsky et al. (19) compared a lower 10 mg/kg/day dose of CBD with the full 20 mg/kg/day in LGS patients. A median 41.9% reduction in drop-seizure frequency was observed in the 20-mg CBD group, while the median reduction was 37.2% in the 10-mg group and 17.2% in the placebo group. Although this study demonstrated patients may gain benefit in seizure reduction by increasing the dose to 20 mg/kg/day, it also displayed an increased risk in AEs. It is generally recommended to begin at 5 mg/kg divided into two intakes a day, then increase to 10 mg/kg/day. If the 10 mg/kg/day dose is well-tolerated and the anti-seizure effect continues, dosing can be increased to the maximum of 20 mg/kg/day (15).

Cannabidiol also proved to effectively reduce seizure frequency at long-term follow-up (20), retaining a consistent reduction (between 42.9 and 44.3%) in seizure frequency at 48 weeks of follow-up. Moreover, 5 out of 104 patients (4.8%) were convulsive seizure-free at 12 weeks of treatment, with more than 40% having a reduction of convulsive seizure frequency ≥50% at each programmed visit of follow-up (18). In terms of median percentage reduction in convulsive seizures, rates of responders, reduction in total seizures, and CGIC-assed improvements, CBD proved greater in the subset of patients concomitantly treated with CLB. Moreover, the combination CBD+CLB showed a benefit in the number of convulsive seizure-free days (16). However, a drug-to-drug interaction increasing levels of active metabolites of both compounds must be assessed and hence CLB dose reduction is recommended if patients experience somnolence or sedation (15, 16).

In conclusion, RCTs settle CBD as a well-tolerated drug, with patients primarily experiencing somnolence, diarrhea, and decreased appetite. The elevation of liver transaminases may be observed mostly in patients on concomitant valproate, and the dose reduction of valproate or CBD is often decisive. The efficacy of CBD on both convulsive and drop seizures is established, with retained efficacy at long-term follow-up. New RCTs in other syndromic or isolated epilepsies populations may widen the field of use of CBD in the next few years.

### Fenfluramine

Fenfluramine (FFA), formerly used at 10 times higher dosage (up to 120 mg/day) as a weight-loss drug, exerts its anti-seizure effect both through the release of serotonin which stimulates multiple 5-HT receptor subtypes, and by acting as a positive modulator of sigma-1 receptors (16, 21–23). Fenfluramine has been approved by the FDA in June 2020 and is currently under evaluation by the European Medicines Agency (EMA). The drug proved significantly effective in reducing seizures in phase-3 trials on DS patients: the 0.8 mg/kg/day treated group did experience a mean 64% reduction in seizures as compared to 34% in the 0.2 mg/kg/day group. Notably, a >75% reduction in seizures occurred in 45% of patients under 0.8 mg/kg/day, in 20.5% of those on 0.2 mg/kg/day compared to 2.5% in the placebo group (23). Fenfluramine has then continued to provide a clinically meaningful reduction in convulsive seizure frequency over a median of 445 days of treatment. The median percent reduction in monthly convulsive seizures frequency was 83.3%. Overall, 62% of patients showed a 50% reduction in convulsive seizure frequency (16).

Together with the anti-seizure effect, FFA has also relatively few drug-drug interactions, primarily a moderate effect on stiripentol (STP), which requires the downward adjustment of FFA dosing to.5 mg/kg/day. No additional interaction with other drugs such as valproate, CLB, and CBD are known (15). The most common AEs reported under FFA treatment include decreases in appetite, weight loss, diarrhea, fatigue, lethargy, and pyrexia (16). The main AEs leading to FFA withdrawal as a weight-loss agent were the occurrence of valvular heart disease (VHD) and pulmonary arterial hypertension (PAH), for which 6-month-echocardiographic monitoring is required together with an ECG. However, at the anti-seizure dosages, no VHD or PAH was observed after a median duration treatment of 256 days. No ECG alterations indicative of atrioventricular conduction or cardiac depolarization alterations were seen, and no mitral or aortic valve regurgitation greater than “trace” was observed in any of the 232 patients with DS who participated in the open-label extension (OLE) study (21, 24, 25).

### Cenobamate

Cenobamate (*Xcopri* or *YKP3089*) is a new ASM that has recently gained approval by the FDA for the treatment of focal-onset seizures in adults. The EMA is currently reviewing the drug for approval as an adjunctive treatment in focal-onset epilepsies. Cenobamate is a tetrazole-derived carbamate compound with a dual mechanism of action; the drug can both enhance the inactivated state of voltage-gated sodium channels, and act as a positive allosteric modulator of the GABA-A receptors, binding at a non-benzodiazepine site (26).

A multicenter, randomized study of patients with uncontrolled focal seizures (27) showed that the adjunctive cenobamate, with dosage groups of 100, 200, and 400 mg/day led to a consistent reduction in focal-seizures frequency after 18-weeks of treatment, with the greatest reduction observed in the 200 and 400 mg/day doses groups. A similar dose-effect relationship was seen when evaluating the responder rates (≥50% in seizure reduction). *Post-hoc* analysis proved seizure frequencies decreased early during cenobamate titration; while, during the 12-week maintenance phase, significantly more patients under the active 200 or 400 mg/day harms achieved seizure freedom as compared to that receiving placebo. Cenobamate is overall well-tolerated, showing mild to moderate severity AEs on the CNS system, such as somnolence, dizziness, and disturbances in gait and coordination, with a linear incidence-dose correlation and disappearance at maintenance. Four cases of hypersensitivity adverse reactions occurred during two RCTs, including one serious AEs of Drug Rash with Eosinophilia and Systemic Symptoms (DRESS) (26, 27). In this case, the rapid titration of 100 mg/week from 200 to 400 mg dose might have contributed to the higher rates of AEs in the 400 mg group; a lower starting dose and a slower titration rate have been shown to reduce the occurrence of hypersensitivity reactions, possibly through the development of immune tolerance (27). As cenobamate inhibits the P450 family cytochrome CYP2C19^*^18, dosing adjustment is needed when adding cenobamate to ASM regimens containing phenytoin or phenobarbital (28); moreover, a dose reduction of CLB should be considered, counteract the increase in plasma levels of desmethylclobazam, its active metabolite. Cenobamate has also been shown to decrease by 25% the plasma exposure to carbamazepine, through the induction of the CYP3A4. Cenobamate could shorten the QT-interval on the ECG in a dose-dependent manner. Hence, cenobamate is contraindicated in patients with familial short QT syndrome, and caution is required in co-administration with other drugs known to reduce the QT interval since a synergistic effect may occur (26, 27). In a short time, data will help to assess cenobamate active time-window on seizures control and real-life data will help to acknowledge whether freedom rates will be borne out in clinical practice. The mechanisms of action and the potential additive or synergistic interactions of cenobamate with concomitant ASMs also warrant further investigation (26).

## Novel Non-Pharmacological Treatments

Neurostimulation comprises different techniques, already implemented in the clinical practice, direct to deliver electrical or magnetic currents to the brain in a non-invasive or invasive way and hence modulating neuronal activity to achieve seizure suppression.

### Vagal Nerve Stimulation

Vagal nerve stimulation (VNS) is approved both in Europe and in the United States as an adjunctive treatment in patients with refractory epilepsies, and it is routinely available in many epilepsy centers, with more than 100,000 patients treated worldwide (6). Vagal stimulation may then turn off seizures originating in regions susceptible to heightened excitability, such as the limbic system, thalamus, and thalamocortical projections. Moreover, an additional mechanism of action derives from the activation of the locus coeruleus and the raphe nuclei, and the regulation of the downstream release of norepinephrine and serotonin, both having antiepileptic effects (29).

Two large RCTs showed VNS efficacy in reducing seizures, achieving a 50% reduction in 31% of patients, and over 50% seizures reduction in 23.4% of the studied population. On the other hand, seizure freedom at long-term follow-up was observed in <10% of patients. Side effects are usually mild and include hoarseness, throat paraesthesia or pain, coughing, and dyspnea. This tends to improve over time or through the adjustment of setting parameters (6).

In conclusion, evidence suggests VNS is well-tolerated in both children and adults with drug-resistant partial epilepsies (30–32); moreover, the newest VNS models can detect ictal tachycardia and automatically deliver additional stimulation to abort seizures or reduce their severity (6).

### Transcutaneous VNS

Developed as a non-invasive alternative to VNS, the transcutaneous VNS (tVNS) acts on the auricular branch of the vagus nerve (ABVN), targeting thick-myelinated afferent fibers in the cymba conchae, and hence activating the ipsilateral nucleus of the solitary tract (NTS) and locus coeruleus. This activation pathway overlaps with the classical central vagal projections, leading to a brain activation pattern similar to that produced by invasive VNS (33). The device consists of a programmable stimulation apparatus and an ear electrode (34). Stimulation setup is adjusted by applying decreasing and increasing intensity ramps and achieving a level just above the individual detection threshold, but clearly below that of pain. Patients usually apply tVNS for 1 h/three times per day (33) and adherence is usually high (up to 88%) (35). Trials converge in demonstrating up to 55% reduction in seizure frequency, with mild or moderate side effects mainly including local skin irritation, headache, fatigue, and nausea (6, 35).

### Deep Brain Stimulation

Deep brain stimulation (DBS) is a minimally invasive neurosurgical technique, which through implanted electrodes can deliver electrical *stimuli* to deep brain structures. Patients with refractory focal epilepsies and not eligible for surgery are usually good candidates (29). Both stimulation of the ictal onset zone and the anterior thalamus have gained approval by the FDA as effective stimulation *sites*, providing a significant and sustained reduction in seizures together with the improvement of the QoL. Nowadays, both DBS and responsive neurostimulation (RNS) are available, being the latter a system able to monitor electrical changes in cortical activity and give small pulses or bursts of stimulation to the brain to interrupt a seizure (36). The interim results of a prospective, open-label, and long-term study did show that the median 60% or greater reduction in seizure frequency is retained over years of follow-up. Moreover, the majority of patients took advantage of treatment with the RNS® System, and 23% experienced at least one 6-month period of seizure freedom (37). The most relevant reported side effects were depressive mood and memory impairment, besides the local side effect of implantation. Nonetheless, it should be stated that RNS is a feasible option in most epilepsy centers in the US, but its use remains limited in other parts of the world. In these cases, DBS could be an option with targets and stimulation parameters selection are largely driven by the experience of the referred center (38, 39).

### Trigeminal Nerve Stimulation

Trigeminal nerve stimulation (TNS) is a novel neuromodulation therapy, designed to deliver high frequencies stimulation in a non-invasive way, hence modulating mood and relieving symptoms in drug-resistant epilepsies. The study of DeGeorgio et al. (40) found that the responder rate (at least 50% reduction in seizures) was 30.2% in the active group, while it was 21.1% in the control group. Moreover, the responder rate did further increase over the 18-week treatment period in the actively treated group. TNS was overall well-tolerated and, when occurring, treatment-related AEs were mild including anxiety (4%), headache (4%), and skin irritation (14%). However, long-term follow-up studies showed inconclusive results (6), meaning further studies and patient monitoring will be needed in the next years.

### Transcranial Direct Current Stimulation

The transcranial direct current stimulation (tDCS) displays the use of two skull electrodes (anode and cathode) to induce widespread changes of cortical excitability through weak and constant electrical currents. Cortical excitability may increase following anodal stimulation, while it generally decreases after cathodal stimulation. Based on this principle, hyperpolarization using cathodal tDCS has been proposed to suppress epileptiform discharges. Major six clinical studies are promising with 4 (67%) showing an effective decrease in epileptic seizures and 5 (83%) exhibiting a reduction of epileptiform activity. However, some results may be misleading due both to the small and heterogeneous nature of the studied populations and to the different setting parameters applied. Hence, nowadays the major achievement is the demonstration that tDCS may be effective and safe in humans; however, further studies will be needed to define setting stimulation protocols and understand the long-term tDCS effectiveness (41).

### Transcranial Magnetic Stimulation

The nerve cells of a brain to a maximum depth of 2 cm can be stimulated using transcranial magnetic stimulation (TMS). To this, low-frequency and repetitive magnetic stimulations have been shown to induce long-lasting reductions in cortical excitability and, hence, have been proposed as a treatment for drug-resistant epilepsies (4). Probably, it is the repeated nature of magnetic pulses which allows modulating the neuronal activity, wherein high frequencies (>5 Hz) would have an overall excitatory effect, while low-frequencies (0.5 Hz) would exert an inhibitory effect on neurons (29).

Despite the optimal stimulation parameters still needing to be clearly defined, they are likely of crucial importance because treatment intensity depends both on the number of pulses and the number of sessions applied over the treatment period. Superior results are achieved in patients with neocortical epilepsy, whit a calculated effect size of 0.71 and 58–80%. This makes sense taking into account the rapid decay of the strength of the magnetic field with distance hence no adequate secondary currents can be elicited in the deep cortex. However, evidence suggests the effects of repetitive TMS may not be restricted to the only site of stimulation but may spread from focal areas to wider areas of the brain.

In conclusion, results should be reproduced in larger cohorts with double-blinded randomized trials, but are promising if compared to the effects currently achieved with invasive neurostimulation techniques for the treatment of epilepsy (42).

## Neuroinflammation and Immunomodulation

Nowadays, the neuroinflammatory pathways are known to contribute to both the development and progression of epilepsy and could be targeted for disease-modifying therapies in epilepsies of wide-range etiologies. Studies on patients with surgically resected epileptic foci have demonstrated inflammatory pathways may be involved, hence the neuroinflammation is not merely a consequence of seizures or brain neuropathology but may induce seizures and brain anatomical damage itself (2).

Finally, any inflammatory response within the brain will be associated with the blood-brain barrier (BBB) dysfunction. Evidence indicates that BBB opening and the subsequent exposure of brain tissue to serum proteins induces upregulation of proinflammatory cytokines and complement system components: this suggests positive feedback between increased brain permeability, local immune/inflammatory response, and neuronal hypersynchronicity (43).

It should also be considered that overall neuroinflammation is a negative disease modifier in epilepsy, however, some inflammatory processes may be involved in tissue repair and brain plasticity after injury hence interference with these beneficial mechanisms should be avoided: anti-inflammatory intervention in the wrong patient and at the wrong time could be ineffective or even harmful. Yet, it is for this reason that evidence remains set at the preclinical level with few reports of use in the clinical practice. The discovery of non-invasive biomarkers of pathological neuroinflammation would enable physicians to identify patients who could benefit from the treatments, also providing a potential marker of therapeutic response.

### IL-1R1-TLR4 Signaling

The Interleukin IL-1R1-TLR4 signaling pathway originates the neuroinflammatory cascade in epilepsy through increased levels of either the endogenous agonists or their receptors, or even a combination of both (2). These findings prompted the clinical use of anakinra, the recombinant, and modified form of the human IL-1Ra protein. Case report studies of Anakinra in patients with intractable seizures did result in a significant reduction of seizure activity and improvement of cognitive skills (44). Moreover, IL-1R1 and TLR4 signaling have been targeted by specific, non-viral, small interfering RNAs (siRNAs) to knock down the inflammasomes or caspase 1 in rats with kindling-induced SE (45).

### Prostanoids

Prostanoids are a family of lipid mediators generated from the cell membrane arachidonic acid by cyclooxygenase enzymes 1 and 2 (COX1 and COX2). Prostanoids bind to specific G protein-coupled receptors (GPCR), hence regulating both innate and adaptive immunity (46).

#### Monoacyl Glycerol Lipase

The monoacyl glycerol lipase (MAGL) is a lipase constitutively expressed by neurons and a key enabler of 2-arachidonoylglycerol (2-AG) hydrolysis. 2-Arachidonoylglycerol is an endocannabinoid, which likewise prostaglandins are involved in seizures genesis. Hence, the upstream inhibition of the MAGL has the potential to be an effective target in epilepsy therapy (2). In 2018 Terrone et al. (47) did demonstrate CPD-4645 (a selective and irreversible MAGL inhibitor) was effective in terminating diazepam-resistant status epilepticus (SE) in mice. Moreover, clinically relevant outcomes such as reduced cognitive deterioration were ensured by CPD-4645 action: reducing post-SE brain inflammation to prevent neural cell damage. Lastly, the authors noted that SE was more promptly stopped in those mice concomitantly receiving the KD, hence suggesting brain inflammation is the common, final, target. Striking inflammation through different inflammatory pathways may enhance neuroprotection and seizure control.

#### COX2 Inhibitors and Prostaglandin Receptor Antagonists

Targeting the inducible enzyme COX2 to that of blocking the prostanoid cascade has been tested to interfere with acute seizures or SE. The importance of timing was demonstrated by early anti-inflammatory interventions showing worsening seizures as compared to late-onset interventions (2, 48). Prostaglandin F_2α_ (PGF), which is anti-ictogenic, is indeed predominant in the first hour after SE onset, then the ratio between PGF and the pathogenic prostaglandin E_2_ (PGE) normalizes in association with an increase in COX2 synthesis (2). Hence, punctual COX2-related treatments have been considered to prevent epileptogenesis and reduce the frequency of seizures in epileptic patients. COX2 inhibition could either be selective (*coxibs* = selective COX2 inhibitors) or non-selective (*aspirin*). In two in-animal studies testing celecoxib and parecoxib over evoked SE, treatment with celecoxib or parecoxib did show to consistently reduce the number and severity of seizures, together with the improvement of spatial memory deficits (2).

Non-selective blockade of COX2 has been also tested in experimental models of epilepsy, and ASA administration over the chronic, latent, epileptic phase could consistently suppress recurrent spontaneous seizures and inhibit the seizure-induced neuronal loss, preventing aberrant neurogenesis in the hippocampus. Thus, ASA is being actively investigated and has the potential to prevent the epileptogenic processes, including SE occurrence, and may avoid pathological alterations in CNS areas (2, 49). Potential cardiotoxicity is the main limit, bordering COX2 inhibition in clinical practice.

Shifting attention downstream to prostaglandin receptors, highly potent PGE receptor (EP2R) antagonists administered from a 4 h-starting point after the onset of pilocarpine-induced SE, proved to mitigate deleterious consequences such as delayed mortality, functional deficits, alterations of the BBB permeability, and hippocampal neurodegeneration (50). The delayed timepoint of administration further brings evidence that EP2R blockade may allow obtaining neuroprotection later in SE stages, mainly reducing long-term sequelae (2).

#### Inflammatory Response Lipid Mediators

Specialized pro-resolving lipid mediators that activate GPCRs have a major role in controlling inflammatory responses in peripheral organs. G protein-coupled receptors activation leads both to reduced expression of pro-inflammatory molecules and increased synthesis of anti-inflammatory mediators which can modulate immune cell trafficking and restore the integrity of the BBB. Neuroinflammation was reduced after the intracerebroventricular injection of the omega-3 (n-3) docosapentaenoic acid-derived protectin D1 (PD1_n−3DPA_) in mouse models of epilepsy. Interestingly, recognition of memory deficits after SE also gained improvements (2, 51). Since PD1_n−3DPA_ derives from n-3 polyunsaturated fatty acids (PUFAs), in humans, it may be possible to non-invasively increase PD1_n−3DPA_ levels through the dietary intake of n-3 PUFAs, which are found in flaxseed, walnuts, marine fish, and mammals (52). Another way may then be the developing stable analogs of pro-resolving lipids (51).

### Oxidative Stress

Activation of the Toll-like receptors (TLRs) can lead to reactive oxygen species (ROS) production, hence promoting and sustaining inflammatory pathways. The detrimental effects of ROS are usually counteracted through the activation of the nuclear factor E2-related factor 2 (Nrf2). Activated Nrf2 translocates to the nucleus where it heterodimerizes with the small Maf proteins (sMaf) and binds to the antioxidant response element (ARE 5′-TGACXXXGC-3′) battery activating transcription of genes that are involved in antioxidant and cytoprotective tasks (53).

Transient administration of *N*-acetyl-cysteine (NAC), a glutathione precursor, did prove to activate Nrf2 in mouse models of SE, thus inhibiting high mobility group box 1 (HMGB1) cytoplasmic translocation in the hippocampal neural and glial cells and preventing the linkage between oxidative stress and neuroinflammation for which the redox-sensitive protein HMGB1 is central (2). Also, high doses (4–6 g/day) of NAC were used in Unverricht-Lundborg disease (ULD), progressive myoclonus epilepsy (PME), showing overall improvement of myoclonus, ataxia, and generalized tonic–clonic and absence seizures. Neuroprotection and improvements in spatial learning abilities were also observed with retained beneficial effects during treatment (54, 55).

Adeno-associated viral (AAV) vectors gene delivery may provide long-term, persistent, induction of Nrf2 expression in a variety of cell types in the brain, with minimal toxicity. The injection of AAV coding for human Nrf2 in the hippocampus of mice with spontaneously recurrent seizures resulted in a reduction in the number and duration of generalized seizures, which interestingly was performed in the already established epileptic phase, highlighting the direct potential of such interventions in the treatment of epilepsy (56).

## Inhibition of P-Glycoproteins

One of the major neurobiological mechanisms proposed to cause drug resistance in epilepsies lays in the removal of ASMs from the epileptogenic tissue through the expression of multidrug efflux pumps such as the P-glycoproteins (P-gps). P-glycoproteins are the final encoded product of the human multi-drug resistance-1 (*MDR-1*) gene, and play a role in treatment response possibly inducing MDR (57, 58). The increased activity of P-gps reduces clinically effective concentrations of ASMs despite adequate serum concentrations, reversing the anti-seizure effects on epileptogenic areas in the parenchyma of the brain (3).

Following the general rule that the higher the lipophilicity of a drug, the faster the entrance into the brain (59), available ASMs are very lipophilic, but more than one-third of the patients do not respond to treatment. The possible reason may be ASMs serve as P-gps substrates; secondly, the P-gps levels are higher (3). Different clinical studies had shown poor prognoses associated with MDR1 gene products, which gave rise to extensive experimental research on the P-gps (3). The adjunctive use of a P-gps inhibitor might counteract drug resistance and efficiently decrease seizure frequency. In addition to verapamil, other first-generation P-gps inhibitors include nifedipine, quinidine, amiodarone, nicardipine, quinine, tamoxifen, and cyclosporin A. It is primarily due to the lack of selectivity and the pharmacokinetic interactions that trials using such agents failed to rule out P-gps inhibition efficacy in other fields such that of oncology (60, 61). First-generation MDR inhibitors required high concentrations to reverse MDR and thus were associated with unacceptable toxicity. In recent years, second and third-generation compounds have been developed which are more selective, highly potent, and non-toxic. Notwithstanding second-generation agents have better tolerability, they still have unpredictable pharmacokinetic interactions (i.e., valspodar is a substrate for cytochrome P450, altering plasma availability of co-administered drugs) and may inhibit other transport proteins. Third-generation inhibitors have more advantages such as high specificity for P-gp, lack of non-specific cytotoxicity, relatively long duration of action with reversibility, and good oral bioavailability. However, despite their selectivity and potency, also this last generation of MDR modulators is far from being perfect and further studies will be needed to outline their effectiveness and safely overcome drug resistance (3, 60). As pertains to clinical research, Iannetti et al. (62) first demonstrated the action of verapamil in a case of prolonged refractory SE and then, subsequently on small series of other types of drug-resistant epilepsies (63, 64).

A novel, yet preclinical, approach for reversing multidrug resistance in epilepsy may derive from the modulation of P-gp by herbal constituents. Nowadays, several herbal formulations and drugs which act by modulating P-gps are available and can be explored as alternative treatment strategies. For example, curcumin (the natural dietary constituent of turmeric) orally administered to pentylenetetrazole-kindled epileptic mice models is known to prevent seizures and related memory impairments (65). The mechanism of action may lie on that curcumin and can reverse multidrug resistance. Hence, curcumin synthetic analogs, which hold more favorable pharmacodynamic properties, have been developed (i.e., GO-Y035); or curcumin has been encapsulated in nanoparticles (NPs) enhancing its solubility and sustaining release inside the brain (66).

Again, piperine (an alkaloid present in black pepper) and capsaicin (the active component of chili peppers) are known to increase curcumin and other P-gps substrates bioavailability and can be therefore used as basic molecules for the development of non-toxic P-gps inhibitors (67, 68).

In conclusion, the identification of an optimal P-gps inhibitor that is potent, effective, and well-tolerated, is desirable to reverse MDR in epileptic patients and will be the challenge of the upcoming years.

## Gene Therapies

Currently lying at the preclinical evidence, gene-based therapy modulates gene expression by introducing exogenous nucleic acids into target cells. The delivery of these large and negatively charged macromolecules is typically mediated by carriers (called vectors) (69). In treating epilepsy, the main hitch is the BBB, which prevents genetic vectors from entering the brain from the bloodstream. Consequently, an invasive approach may be needed (29). Moreover, several considerations need to be taken into account when translating gene therapy into clinical practice, namely the choice of the viral vector, promoter, and transgene (6).

### Viral Vectors

Viral gene therapy may employ three classes of viral vectors, namely, adenovirus (AD), adeno-associated virus (AAV), and lentivirus. All these three viral vectors have successfully demonstrated to attain high levels of transgene delivery in *in-vivo* disease models and clinical trials. However, the risks of immunogenic responses and transgene mis-insertions, together with problems in large-scale production are still a deal to face (70).

Adeno-associated viruses belong to the Parvoviridae family and proved to retain favorable biology, leading their recombinant forms (rAAVs) to become the main platform for current *in-vivo* gene therapies (29). A limited clinical trial on patients with late-infantile neuronal ceroid lipofuscinosis (LINCL) did prove neurosurgical gene therapy to be practical and safe, supporting the potentialities of this kind of approach (71). However, in the view of removing invasiveness, interest was moved to engineered capsid which can confer the ability to cross the BBB and transduce astrocytes and neurons, allowing direct intravenous injection. This was achieved through a process of directed selection in a mouse strain, and further work would be needed to develop a similar variant for use in humans (6, 72).

Retroviruses such as lentivirus share with AAVs the ability to infect neurons and lead to a stable expression of transgenes. Lentiviral vectors (lentivectors) are RNA viruses and the transgenes can integrate into the host genome through the reverse transcriptase gene. However, possible insertional mutagenesis may be reduced by using integration-deficient lentivectors, which simultaneously ensure stable transduction (73). Lastly, lentivectors can package larger genes or regulatory elements as compared to AAVs (6).

Different viral vectors intrinsically tend to infect different neuronal and glial subtypes, but the high specificity of the target is far from their properties. Hence, several efforts have been made that to identify specific neuron-type targeting promoters: the calcium/calmodulin-dependent protein kinase II (CamKII) promoter is suitable to manipulate excitatory neurons in the forebrain; on the other hand, targeting inhibitory interneurons may be difficult as promoters for specific GABAergic neurons are poorly defined (6). Finally, the optimal promoter should provide the expression of a level of transgene which is sufficient to moderately alter cell properties but avoids cytotoxicity (6, 74).

As for the transgene, gene therapies have been commonly built on the basis that the excitation–inhibition balance is altered in epilepsy. Hence, on a general principle, gene therapy may work through modulating the expression of neuropeptides, and regulation of the neuropeptide Y (NPY) did already show promise, acting both on pro-excitatory Y1 and pro-inhibiting Y2 receptors in the hippocampus (6, 75). Another way may be that of regulating potassium channels; overexpression of the potassium channel Kv1.1 proved effective in preventing epileptogenesis in a mouse model of focal epilepsy, the physiological basis may lie on the modulation of both neuronal excitability and neurotransmitter release (76, 77). Lastly, *chemogenetics* refers to the possibility to use gene transfer to express receptors that are insensitive to endogenous neurotransmitters but highly sensitive to exogenous drugs, in a receptor-to-drug therapeutic approach. This promising approach will also allow adjusting the activating drugs to find the optimum dosage with low interference with normal brain function but efficiently suppressing seizures (6). Further refinements of *chemogenetics* have jet got underway, which may use receptors detecting out-of-range extracellular elevations of the concentration of glutamate and, therefore, inhibiting neurons, preventing drug administration. Although attractive, this strategy will need further work to assess the risk of immunogenicity (6).

### Non-viral Strategies

Some of the issues of viral vector-based gene therapy may be overcome by non-viral gene strategies, which provide advantages with regards to the safety profile, localized gene expression, and cost-effective manufacturing. Non-viral gene delivery systems are engineered complexes or NPs composed of the required nucleic acid (pDNA or RNAs) and other materials, such as cationic lipids, peptides, polysaccharides, and so on (70). These vectors have low production costs, can be topically administered, can carry large therapeutic genes, use expression vectors (such as plasmids) that are non-integrating, and do not elicit detectable immune response also after repeated administrations (29, 70). Cationic lipid-based vectors are currently the most widely used non-viral gene carriers. Limitations may include low efficacy due to the poor stability and rapid clearance, or the possible generation of inflammatory or anti-inflammatory responses. Hence, cationic polymers, such as poly(L-lysine) (PLL) or modified variants (PEGylated PLL), constitute alternative non-viral DNA vectors that are attractive for their immense chemical diversity and their potential for functionalization (69).

### Antisense Oligonucleotides Therapies

Oligonucleotides are unmodified or chemically modified single-stranded DNA sequences (of up to 25 nucleotides) that hybridize to specific complementary mRNAs. Once bound to targeted mRNAs, oligonucleotides can either promote RNA degradation or prevent the translational machinery through an occupancy-only mechanism, referred to as *steric blockage*. Anyhow, the process leading to protein formation is inhibited. Synthesizing antisense oligonucleotides (ASOs) must deal with making a structure that must be suitable for a stable and selective oligonucleotide/mRNA complex. Moreover, oligonucleotides are rapidly degraded by endo- and exonucleases and the mononucleotides products may be cytotoxic (29, 78). Hence, the use of ASOs in clinical practice requires overcoming problems related to the design, bioavailability, and targeted delivery (78). To date, few *in-human* studies have been conducted that primarily addressed invariably progressive and fatal diseases such as PMEs (79, 80). The authors proved the feasibility of the ASOs-based approach by specifically customizing oligonucleotides over the genetic defect of patients. This opens the way to N-of-1 trials, which will hopefully be the road of the next few years not only in oncology but also in epileptic patients (81).

## Stem Cell Therapy

Recurrent seizures are associated with the loss of inhibitory GABAergic interneurons. Herby, the replacement of lost interneurons through grafting of GABAergic precursors might improve the inhibitory synaptic and reduce the occurrence of spontaneous seizures (6).

Currently, in a pioneering way, progenitors from the medial ganglionic eminence (MGE) derived either from fetal brains or, to avoid the need for immune suppression, from human induced pluripotent stem cells (hiPSCs) proved the most suitable for treating epilepsy, particularly with temporal lobe onset features. Medial ganglionic eminence cells show pervasive migration, differentiate into distinct subclasses of GABAergic interneurons, and efficiently get incorporated into the hippocampal circuitry improving inhibitory synaptic neurotransmission (82, 83). An important point is that MGE progenitors from fetal brains hoist ethical issues, and it is also a challenge to obtain the adequate amount of cells required for clinical application (82). Consequently, the MGE progenitors derived from hiPSCs appear the most suitable donor cell type, as they do not raise ethical problems and are also compatible with patient-specific cell therapy in non-genetic epileptic conditions. However, it will be important to understand whether the suppression of spontaneous recurrent seizures is transient or enduring after the GABAergic progenitor cells grafting (82); moreover, it will be important to assess the safety profile of these hiPSCs, hence they may either exhibit genomic instability or cause undesired differentiation raising concerns for *in human* application (6). In conclusion, the results are exciting, but some points need to be addressed in the next years, before starting a true *in human* application.

## Conclusions

A variety of drugs are being investigated for the treatment of epilepsy, many of whom target previously neglected pathophysiological pathways but demonstrate a favorable efficacy profile, together with low to mild grade AEs (15). Traditional ASMs, given alone or in a fair combination, are invariably the initial therapeutic approach; afterward, if drug resistance occurs, more than one underlying pathophysiological mechanism may likely contribute (14). Currently, uncontrolled epilepsy is often disabling, with patients experiencing increased comorbidity, psychological, and social dysfunction, combined with an increased risk of premature death. In younger patients, cognitive and neurodevelopmental impairments are severe consequences of recurrent spontaneous seizures, impacting the QoL and future independence (44). Accordingly, gaining a reduction of either the severity or frequency of seizures might have benefits (44) and hitherward new therapeutical strategies are in the pipeline.

Cannabidiol, FFA, and cenobamate have been shown to efficiently control seizures and are generally well-tolerated; particularly, an increase in the number of seizure-free days was observed with positive outcomes on the QoL of patients (16). Comparison of treatments such as VNS, DBS, and TNS are needed to decide which modality is the most effective; moreover, data collection on promising *non-invasive* neurostimulation modalities will allow getting a precise estimate of their therapeutic efficacy and long-term safety (30) (Tables 1, 2).

**Table 1:** Advanced RCTs on new drugs for epilepsy treatment.

**References**	**Type of RCT and treatment**	**Study population (n**°** of pts, type of epilepsy, mean age ± SD)**	**Previously tested vs. concomitant ASMs (n**°**)**	**Primary end point**	**Outcomes**
Devinsky et al. (17)	Double-blind, placebo-controlled RCT 20 mg/kg/d CBD oral solution	214 DS pts M 9.8 ± 4.8 years	4.0 3.0	Change in CSF	- 38.9% reduction in CSF in the CBD group vs. 13.3% in reduction in the placebo group - ≥50% reduction in CSF in 43% pts in the CBD group vs. in 27% pts in the placebo group - 5% pts sz-free in the CBD group vs. 0% sz-free in the placebo group
Thiele et al. (18)	Double-blind, placebo-controlled, phase 3 RCT 20 mg/kg/d CBD oral solution	171 LGS pts M 15.4 ± 9.25 years	6.0 3.0	Change in monthly frequency of drop sz	- 43.9% reduction in monthly drop sz frequency in the CBD group vs. 21.8% reduction in the placebo group - ≥50% reduction in drop sz frequency in 44% pts in the CBD group vs. in 24% pts in the placebo group - Improved overall condition in 58% pts in the CBD group vs. in 34% pts in the placebo group
Devinsky et al. (19)	Multicenter, double-blind, placebo-controlled, phase 3 RCT 10 or 20 mg/kg/d CBD oral solution	225 LGS pts M 15.6 ± 9.9 years	6.0 3.0	Average change in drop sz frequency	−41.9% reduction in drop sz frequency in the 20 mg CBD group vs. 37.2% reduction in the 10 mg group vs. 17.2% reduction in the placebo group - 50% reduction in drop sz frequency in 39% pts in the 20 mg CBD group vs. in 36% pts in the 10 mg group vs. in 14% pts in the placebo group - Improved PGIC in 57% pts in the 20 mg CBD group vs. in 66% pts in the 10 mg group vs. in 44% pts in the placebo group
Devinsky et al. (20)	OLE 20 up to 30 mg/kg/d CBD oral solution	264 DS pts M 9.8 ± 4.4 years	na 3.0	Long-term safety and tolerability of CBD	- 37.5% reduction in CSF retained for up to 48 w; - 4.8% pts were convulsive sz free and 2.9% pts were totally sz-free in the last 12 w of treatment - ≥50% reduction in CSF observed in more than 40% of pts - 93.2% of pts reported AEs: 36.7% mild; 39.0% moderate; 29.2% severe
Lagae et al. (23)	Double-blind, placebo-controlled RCT 0.2 or 0.7 mg/kg/d of fenfluramine HCl oral solution	119 DS pts M 9.0 ± 4.7 years	na M 2.4 ± 1.0	Change in monthly CSF	- 74.9% reduction in CSF in the 0.7 mg/kg/d group vs. 42.3% reduction in the 0.2 mg/kg/d group vs. 19.2% reduction in the placebo group - ≥50% reduction in CSF observed in 68% pts in the 0.7 mg/kg/d group vs. in 38% pts in the 0.2 mg/kg/d group vs. in 12% pts in the placebo group - 8% pts were sz-free in the 0.7 mg/kg/d group vs. 8% in the 0.2 mg/kg/d group vs. 0% in the placebo group - Improved CaGI in 55% pts in the 0.7 mg/kg/d vs. in 41% pts in the 0.2 mg/kg/d vs. in 10% pts in the placebo group
Lai et al. (25)	OLE 0.2 up to 0.7 mg/kg/d of fenfluramine HCl oral solution (up to 0.4 mg/kg/d if concomitant STP)	232 DS pts M 9.1 ± 4.7 years	na na	Number of pts with VHD or PAH during treatment (median 256 d)	- No pts developed VHD or PAH - 23% pts showed trace of mitral regurgitation (mostly transient)
Krauss et al. (27)	Multicentre, double-blind, placebo-controlled, dose-response RCT 100–200–400 mg/d cenobamate oral solution	437 pts with drug-R focal epilepsy M 39.8 ± 11.8 years	2.0–3.0 2.0–3.0	Change in monthly focal sz frequency	- 55.0% reduction in focal sz frequency in the 200 and 400 mg/d group vs. 35.5% reduction in the 100 mg/d group vs. 24.0% reduction in the placebo group - ≥50% reduction in sz frequency observed in 64% pts in the 400 mg/d group vs. in 56% pts in the 200 mg/d group vs. in 40% pts in the 100 mg/d group vs. in 25% pts in the placebo group
Sperling et al. (28)	Multicenter, ongoing, phase 3, OLE 12.5 up to 400 mg/d cenobamate oral solution	1,339 pts with drug-R focal epilepsy M 39.7 ± 12.84 years	2.0–3.0 2.0–3.0	Long-term safety of cenobamate	- At least one AE was reported in 84.2% of pts: 77.8% were mild-moderate - At least one serious AE was reported in 8.1% of pts: seizures; pneumonia; fall; dizziness - No cases of DRESS were identified when starting at low dose and titrating every 2 w

*AEs, adverse events; ASMs, antiseizure medications; BDI, beck depression inventory; CaGI, caregiver global impression; CBD, cannabidiol; CSF, convulsive seizure frequency; d, day; drug-R, drug-resistant; DS, Dravet syndrome; LGS, Lennox-Gastaut syndrome; LINCL, late infantile neuronal ceroid lipofuscinoses; M, mean; n°, number; na, not assessed; OLE, open label extension; PAH, pulmonary arterial hypertension; PGIC, patient global impression of change; Pts, patients; RCT, randomized clinical trial; Ref, reference; SD, standard deviation; SF, seizure frequency; SUDEP, sudden unexpected death in epilepsy; STP, stiripentol; sz, seizures; VHD, valvular heart disease; w, weeks; y, years*.

**Table 2:** Advanced RCTs on new non-pharmacological treatments for epilepsy.

**References**	**Type of RCT and treatment**	**Study population (n**°** of pts, type of epilepsy, mean age ± SD)**	**Previously tested vs. concomitant ASMs (range, mean)**	**Primary end point**	**Outcomes**
Orosz et al. (31)	Retrospective, open-label, multicenter study VNS Therapy, Cyberonics	347 pts with DRE of any type M 2.7 ± 3.0 y	1–27, M 6.9 1–6, M 3.0	Change in the “predominant sz type” frequency at 12 months of FU	- 5.5% pts became sz free (i.e., no sz of the “predominant sz type”) - 32.1% pts achieved ≥50% sz reduction - 17.1% pts had a 25–49% sz reduction - The percentage of responders increasing over time: 32.5%, 37.6%, and 43.8% at 6, 12, and 24 months of FU
Boon et al. (32)	Prospective, observational, unblinded, multicenter study Model 106 VNS Therapy System	31 pts with focal-onset sz, iTC, and DRE M 39.6 ± 13.4 y	na na	≥80% sensitivity for iTC sz in at least one CBSDA, and investigate FP rate	- 37/66 (56%) sz were associated with a ≥20% heart rate increase - 11/66 (17%) sz were associated with iTC (55% or 35 bpm heart increase from baseline, minimum 100 bpm) - ≥80% sz detection sensitivity achieved in multiple CBSDA - FP rate ranged from 0.5 to 7.2/h
Bergey et al. (37)	Prospective, open-label, multicenter study RNS System, NeuroPace	230 pts with focal-onset sz, sGTC sz, and DRE (feasibility and pivotal studies already completed) M 34.0 ± 11.4 y	na 0–8, M 2.9	Long-term efficacy and safety of RNS	- 66% median reduction in sz at 6 y of FU with a RR of 56% - Improvements in QoL were maintained at 5 y of FU (*p* < 0.05) - Most common serious device-related AEs (5.4 y of FU) were implant site infection (9.0%) and neurostimulator explantation (4.7%)
DeGiorgio et al. (40)	Double-blind, parallel-group, phase 2, multicenter RCT External pulse generator for eTNS	50 pts with focal-onset sz, sGTC sz, and DRE M 33.7 y	na, M 3.35 na	Change in mean monthly SF, and RR (>50% sz reduction), time to the fourth sz	- 16.1% reduction in sz frequency for the treatment group vs. 10.5% reduction for the control group - 30.2% RR for the treatment group vs. 21.1% RR for the control group - Net increase 2.5 d (20%) to fourth sz in the treatment group vs. decrease 5 d (21.7%) in the control group (*p* = 0.73)

*AEs, adverse events; ASMs, antiseizure medications; bpm, beats per minute; CBSDA, cardiac-based seizure detection algorithm; DRE, drug-resistant epilepsy; eTNS, external trigeminal nerve stimulation; FP, false positive; FU, follow-up; h, hours; iTC, ictal tachycardia; M, mean; n°, number; na, not assessed; Pts, patients; RCT, randomized clinical trial; Ref, reference; RNS, responsive neurostimulation; RR, retention rate; SF, seizure frequency; sGTC, secondarily generalized tonic-clonic; sz, seizures; VNS, vagal nerve stimulation; y, years*.

Evidence on the role of neuroinflammation in epilepsy suggests that drugs that modulate specific inflammatory pathways could also be used to control seizures and improve neurological comorbidities, such as cognitive deficits and depression. Notably, many anti-inflammatory drugs are already available and could be repurposed in patients with epilepsy. Another mechanism likely involved in drug-resistant epilepsies is the undue expression of multidrug efflux transporters such as P-gps (52); however, the use of P-gps inhibitors in the clinical practice did prove disadvantageous for inseparable systemic toxicity (3). This arises the need to directly modulate not the transport but the expression of the P-gps (3). Finally, epilepsy represents a field suitable for the development of personalized approaches, requiring integration of clinical measures with both genomics and other *-omics* modalities (14).

**Figure 1:**
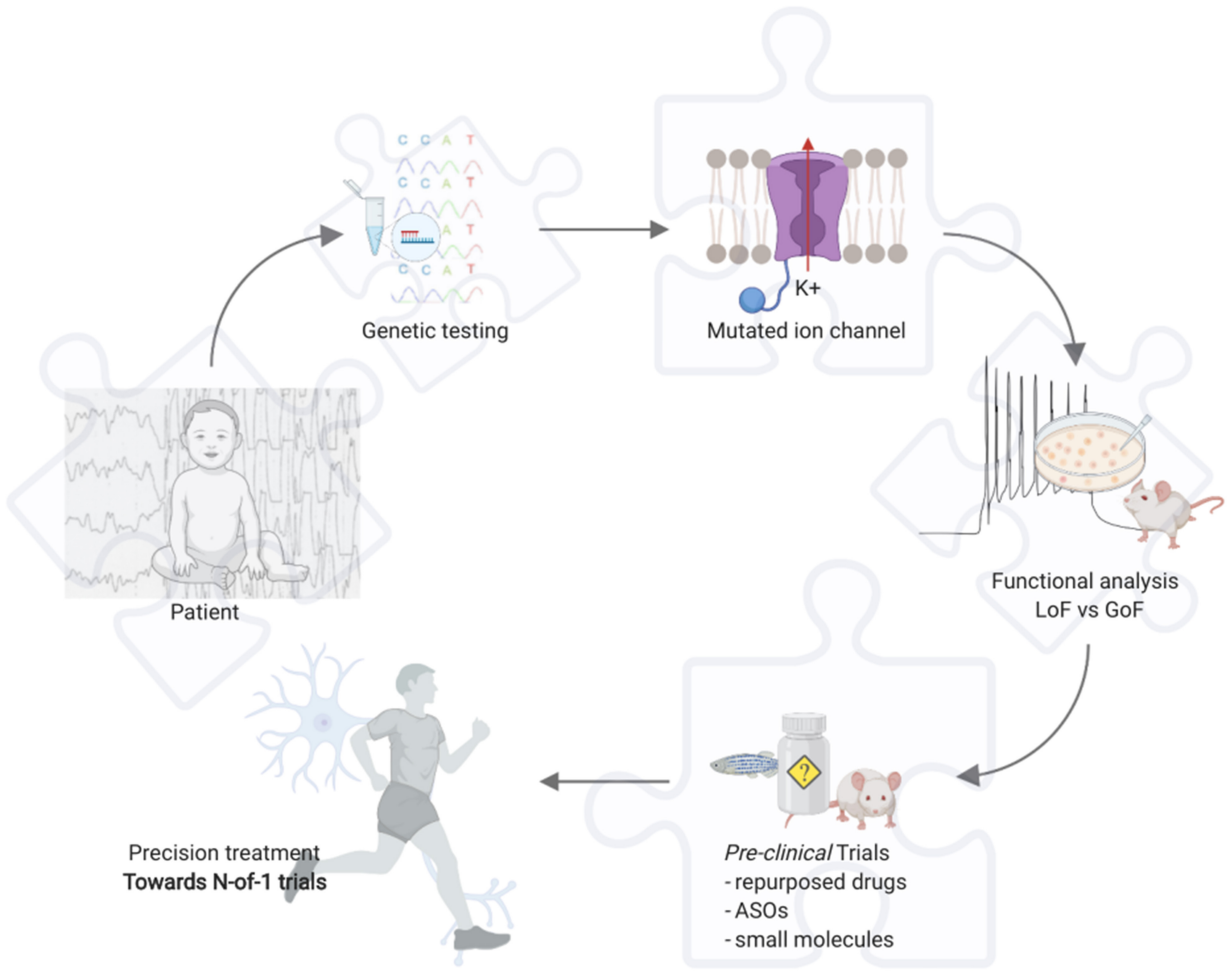
Example for precision medicine in epileptic channelopathies. Toward N-of-1 trials. Created with BioRender.com. ASOs, antisense oligonucleotides; GoF, gain of function; LoF, loss of function.

Today epilepsy carries restrictions in the everyday life of the affected people, together with social burdens, and eventually high-level burdens for caregivers in EE. Hitherward, the continuous pursuit of the best treatment approach that nowadays, with the widening understanding of the pathophysiological basis of the epilepsies, is inevitably moving toward a “*precision*” approach. Gene hunting and new genes discovery proved essential in this way, but further support derives from functional *in-vitro* and *in-vivo* studies, i.e., in epileptic channelopathies it is crucial to understand whether the phenotype is caused by the loss- or gain-of-function mutations in the encoded protein through *patch-clamp* studies (Figure 1). Likewise, if a novel gene is identified it is fundamental to understand through which mechanism it may cause the disease, consequently identifying the best treatment to reverse the functional defect. However, given a PM-based approach, this may not yet be enough, and a holistic evaluation of the patient involves the clinician to deeply know an own expected vulnerability to drugs through *pharmacogenomics*; thus, avoiding potential AEs.

Targeting the biological mechanism responsible for epilepsy could lead either to repurpose as ASMs and adjust dosages of drugs yet used in other fields of medicine (i.e., FFA, COX2 inhibitors, or inhibitors of P-gps) or even to develop outstanding treatments such as gene therapy. Great advances have been achieved in gene-based therapies, ranging from the development of new delivery material to the improved potency and stability of delivered nucleic acids. However, this field is still actually limited by the little understanding of exogenous-endogenous DNAs interaction and the invasive nature of some neurosurgical approaches. Moreover, targeted approaches (i.e., gene therapy, but also innovative drugs) currently carry high economic costs, which are covered by pharmaceutical industries during clinical trials but are hardly affordable for patients. In the new few years, the standardization of drug development, together with a larger use, and faster approval by regulatory agencies will probably make these treatments cheaper for patients.

The inflammatory pathways are common over epilepsies of different etiology and may therefore be reliable targets for treatment. However, targeting such complex and cross-interacting pathways of the human system may prove difficult, potentially altering basic life signals and causing a *plethora* of AEs further impacting the QoL of patients. Hence, also from this site, the next few years will be important to expand our knowledge and act consciously or even early, having fully comprised the red flags (biomarkers) of altered pathways through *-omics* studies.

Overall, research has changed our approach to epileptic patients, but PM is not always straightforward, and the pathophysiology of diseases may be more complex than what we can *model*, as different concomitant genetic variants, epigenetics, or the environment may modulate phenotypes in unintelligible and irreproducible ways. Moreover, nowadays patients are still often belatedly diagnosed raising the need to better define the way clinicians address *phenotyping*, which if incomplete could lead primarily toward the application of NGS epilepsy panels and then to whole-exome or genome sequencing, but invariably delaying diagnosis. Hence, also newer and standardized means of *phenotyping* will be needed, and wide opportunities in this are opened by the human phenotype ontology (HPO), a standardized vocabulary to describe phenotypic abnormalities. The hope will remain that of early diagnosis, early and non-invasive treatment to heal symptoms, improving the QoL of patients, and, in encephalopathies, improving the learning curve of patients.

## Author Contributions

AR: conceptualization, writing-original draft, writing-review, and editing lead. AG: writing-original draft. GB: writing-review and editing support. EA, MV, GP, MI, SL, VS, and CM: writing-review and editing support. PS: conceptualization, funding acquisition, supervision, writing-review, and editing. All authors contributed to the article and approved the submitted version.

## Funding

This work was developed within the framework of the DINOGMI Department of Excellence of MIUR 2018-2022 (legge 232 del 2016).

## Conflict of Interest

AR has received honoraria from Kolfarma s.r.l and Proveca Pharma Ltd. PS has served on a scientific advisory board for the Italian Agency of the Drug (AIFA); has received honoraria from GW Pharma, Kolfarma s.r.l., Proveca Pharma Ltd., and Eisai Inc., and has received research support from the Italian Ministry of Health and Fondazione San Paolo. The remaining authors declare that the research was conducted in the absence of any commercial or financial relationships that could be construed as a potential conflict of interest.

## Publisher's Note

All claims expressed in this article are solely those of the authors and do not necessarily represent those of their affiliated organizations, or those of the publisher, the editors and the reviewers. Any product that may be evaluated in this article, or claim that may be made by its manufacturer, is not guaranteed or endorsed by the publisher.
